# Taking modern psychiatry into the metaverse: Integrating augmented, virtual, and mixed reality technologies into psychiatric care

**DOI:** 10.3389/fdgth.2023.1146806

**Published:** 2023-03-24

**Authors:** T.J. Ford, Derrick M. Buchanan, Azeezat Azeez, David A. Benrimoh, Irakli Kaloiani, Igor D. Bandeira, Saron Hunegnaw, Lucy Lan, Mia Gholmieh, Vivek Buch, Nolan R. Williams

**Affiliations:** ^1^ Brain Stimulation Laboratory, Department of Psychiatry and Behavioral Sciences, Stanford University, Palo Alto, CA, United States; ^2^ Department of Psychiatry, McGill University, Montreal, QC, Canada; ^3^ Neurosurgery, Stanford University, Palo Alto, CA, United States

**Keywords:** metaverse web 3.0, psychiatry 3.0, medical education, brain stimulation, biofeedback, equity, ethics

## Abstract

The landscape of psychiatry is ever evolving and has recently begun to be influenced more heavily by new technologies. One novel technology which may have particular application to psychiatry is the metaverse, a three-dimensional digital social platform accessed via augmented, virtual, and mixed reality (AR/VR/MR). The metaverse allows the interaction of users in a virtual world which can be measured and manipulated, posing at once exciting new possibilities and significant potential challenges and risks. While the final form of the nascent metaverse is not yet clear, the immersive simulation and holographic mixed reality-based worlds made possible by the metaverse have the potential to redefine neuropsychiatric care for both patients and their providers. While a number of applications for this technology can be envisioned, this article will focus on leveraging the metaverse in three specific domains: medical education, brain stimulation, and biofeedback. Within medical education, the metaverse could allow for more precise feedback to students performing patient interviews as well as the ability to more easily disseminate highly specialized technical skills, such as those used in advanced neurostimulation paradigms. Examples of potential applications in brain stimulation and biofeedback range from using AR to improve precision targeting of non-invasive neuromodulation modalities to more innovative practices, such as using physiological and behavioral measures derived from interactions in VR environments to directly inform and personalize treatment parameters for patients. Along with promising future applications, we also discuss ethical implications and data security concerns that arise when considering the introduction of the metaverse and related AR/VR technologies to psychiatric research and care.

## 1. Introduction and Background

Virtual reality and augmented reality are emerging technologies with significant potential to effect psychiatric diagnosis, treatment, research, and training (1). Virtual reality (VR) involves immersion of the user within an interactive, computer-generated simulation enabled by a headset; users can receive visual, auditory, and sometimes olfactory and haptic sensations from the environment to increase the feeling of truly being immersed in this 3D digital world (1, 2). Augmented reality (AR) supplements the real-world environment by overlaying digitally-created images atop real-world images (e.g., Snapchat) (3). Mixed reality (MR) refers to real-world and virtual objects interacting with each other within the immersive space, allowing users to have greater control over the virtual objects than pure augmented reality (4). AR, VR, and MR are also referred to as extended reality (XR) or the metaverse, aka “Web 3.0” (5–7). There is a certain amount of flexibility in terminology and definitions as this field continues to emerge and integrate into widespread use.

The concept of VR was first formulated in the 1950's, with initial uptake within the entertainment industry, especially gaming and business, as a unique way to market products (8). In the last few decades, VR and increasingly AR have found applications within healthcare, such as post-stroke rehabilitation, pre-operative planning, image-guided surgery, medical anatomy education, pain reduction, and even as cognitive reserve training for patients suffering from early to moderate Alzheimer's disease (2, 5, 9–12). Currently, more than 230 companies are producing various products related to VR, including large technology corporations such as Meta, Apple, Microsoft, Google, and Samsung, and increasingly pharmaceutical companies (ex. Orion Corporation in Finland using VR for chronic pain) looking to compete within the emerging digital therapeutics sphere (2). Startups, such as Applied VR and Amelia Virtual Care, offer software/hardware packages to medical providers enabling integration of virtual reality into patient care (13, 14). Applied VR received US FDA breakthrough device designation and marketing authorization in 2021 for its product RelieVRx, a prescription-use VR system which utilizes cognitive behavioral therapy (CBT) to treat chronic low back pain over the course of 8 weeks. XR is becoming increasingly recognized as a legitimate adjunctive medical treatment supported by a growing base of research; however, XR as an efficacious treatment modality continues to need more rigorously-designed double-blinded, randomized clinical trials (15). Such clinical trials will also be imperative for establishing potential risks and adverse events that may result from using XR. The long-term effects of XR on the body and mind are still unknown, but short-term adverse events like eye strain, cyber sickness (akin to motion sickness), and reality distortion have been reported (16, 17).

Within mental health, VR has been studied since the 1990's, when Rothbaum et. al, 1995 conducted the first VR study on acrophobia within college students, ultimately spawning a new domain of research establishing the efficacy of VR exposure therapy for anxiety and related disorders (2, 18). There have been over 127 VR intervention clinical studies conducted on anxiety disorders, including: specific phobias, social anxiety disorders, PTSD, and panic disorder (with and without agoraphobias) using increasingly sophisticated VR-based CBT (15, 19). Systematic reviews and meta-analyses show that VR therapy works more effectively than imaginal therapy, as many people experience difficulty with visualization, and as effectively as *in vivo* exposure therapy (20, 21). VR has expanded to include more than 8 studies on psychotic disorders, ranging from applying VR-based CBT to improve cognition and social skills, to using personalized avatars matched to a patient's persecutory auditory hallucination to encourage empowering dialogue (15). VR has also been studied, albeit less commonly, within substance use treatment, eating disorders, forensic psychiatry, and depression treatment (15).

VR has the possibility of making mental health treatment available to more patients, improve cost-effectiveness of therapy, and potentially be a highly efficacious treatment adjunctive to medications, therapy, and procedural psychiatry (15). The opportunity to incorporate VR into psychiatric medical education is also nascent and emerging. Aakhus et. al, 2020 pioneered a VR-based ECT training to standardize learning and provide trainees with opportunities for multiple simulation repetitions (22). Given the great shortage of mental health practitioners, especially within the emerging subspecialties of interventional psychiatry, as well as relative lack of standardization within current training protocols for psychiatric procedures, VR-based education could potentially streamline and standardize competencies in this area.

Implementation of XR within regular medical and specifically psychiatric clinical practice has yet to progress much (15). Within a minority of academic research universities, some research groups have enabled the concurrent development of clinical VR practices and educational dissemination of related material. At Stanford University, psychiatrist and professor Dr. Kim Bullock runs the Virtual Reality and Immersive Technology Clinic, treating phobias, PTSD, OCD, social anxiety, and other disorders *via* VR-based therapy, as well as an educational podcast “Psychiatry XR” (23, 24). The Stanford Neurosurgery team utilizes VR for training, teaching, and surgical preparation to improve patient experiences (25). Dr. Jeremy Bailenson studies the psychology within XR in his Virtual Human Interaction Lab and has pioneered the first course where students are taught entirely within VR (26, 27). Other universities, such as University College of London, Oxford, and University of Southern California also offer robust academic research and emerging XR clinical care. There are increasingly more therapists within community mental health who are venturing into VR to help deliver talk therapies. However, to further advance XR clinical implementation, the field will need to: demonstrate the added value of cost-effectiveness and efficacy; implement more high-quality and replicable research studies; widen treatment indications; and address training gaps in XR setup/delivery, technical obstacles, and high costs (15).

## 2. Medical education in the metaverse

The increasing usage of AR/VR in the past decades for medical education has been substantial (28). Interventions based on these tools could assist medical students in simulating physician roles, such as collecting previous medical history, conducting physical exams, generating diagnostic hypotheses, and proposing therapeutic management of several illnesses. Especially for rare diseases that medical students, residents, and fellows are less likely to encounter (29), augmented reality technology could be an informative, immersive training medium. Utilizing the metaverse and XR could also create learning opportunities to promote clinical knowledge enhancement and protect patient safety, reducing risks and ethical concerns related to exposing individuals to healthcare professionals still under training (30). Moreover, there may be ways that XR could enhance current methods of medical training, or lead to the creation of brand new, previously unforeseen training opportunities (ex. “Taking” someone inside of a VR synapse and watching cellular interactions from the perspective of a neurotransmitter: a la Magic School Bus). That being said, we're not suggesting that XR training could or should replace traditional standard training protocols, but that it is a tool worthy of exploration and utilization.

Several role-playing training technologies using avatars have been used in different medical specialties, such as neurology, internal medicine, and general surgery (31). Considering that psychiatry is a medical specialty in which physical examination is not the primary tool in generating diagnostic hypotheses (32), psychiatry is a promising use-case for virtual reality tools, as psychiatric training in general would not suffer from the absence of physical contact between trainees and the patient as has largely been demonstrated through the shift into video/tele-conference based psychiatry (33) (though exceptions do exist, for example in the diagnosis and treatment of neuropsychiatric illness such as functional neurological disorder). Previous attempts to use digital gaming and online resources in psychiatry may serve as inspirations for the potential utility of XR/metaverse applications in psychiatric teaching. For example, in 2006, an inpatient psychiatric unit was built using Second Life—an online video game that simulates life in a built world—with the aim of assisting trainees in their understanding of psychosis which ultimately increased participants' understanding of visual and auditory hallucinations (34). This intervention suggests that integrating these new tools could promote many possible learning avenues in medical education and for the general public. Other mental disorders with potential clinical application within the metaverse and AR/VR are attention-deficit/hyperactivity disorder (4), autism spectrum disorder (35), anxiety disorders and specific phobias (18, 20), and posttraumatic stress disorder (36).

Furthermore, traditionally, procedure-based treatments have been limited in psychiatry, with electroconvulsive therapy and more recently repetitive transcranial magnetic stimulation (rTMS) being the two most commonly practiced procedures, usually in the context of specialized services. However, with the development of new neurotechnologies and the rise of interventional psychiatry as a subspecialty in the field (37), there will be a growing need to train more psychiatric trainees in such procedures and disseminate procedure training more widely. To that end, educational training in the metaverse could all be recorded, enabling users to rewatch their training to correct mistakes, and even to disseminate these videos to an unlimited number of trainees simultaneously (ex. An unlimited number of trainees could view a single patient interaction simultaneously). More specific to brain stimulation, AR and VR may prove to be helpful in training practitioners in non-invasive brain stimulation techniques, such as transcranial direct current stimulation (tDCS) or more advanced forms of transcranial magnetic stimulation (TMS), such as the novel Stanford Neuromodulation Therapy (SNT) protocol which requires more precise coil targeting (38). Medical students, residents, fellows, and even attendings who would like to expand their skill range, could use AR/VR tools to learn how to achieve more precise positioning of noninvasive neuromodulation devices on the head. These technologies could facilitate training by providing the opportunity to generate reports analyzing performance to provide trainees with feedback, and this feedback could likely be personalized, facilitating efficient, comprehensive skill acquisition (28). Moreover, exposing trainees to AR/VR content of the most common side effects of these techniques could create opportunities to enhance therapeutic skills in a risk-free decision-making environment—this is especially relevant given that many of these side effects, such as seizures during rTMS, are quite rare (39) and therefore unlikely to be encountered during real-life training. However, carefully designed controlled trials will be needed to provide data if these methods are efficacious in delivering superior teaching strategies.

## 3. Non-Invasive brain stimulation in the metaverse

A key question that must be asked when considering the potential impact of the metaverse on psychiatric care is what, precisely, would be *different* about metaverse-enabled technologies compared to currently available technology (40). That is, what would be the unique impact of the metaverse beyond what is currently possible with teleconferencing or virtual reality systems? When considering this issue, one therapeutic area which seems to be well-positioned to benefit in novel ways from the metaverse is brain stimulation, such as repetitive transcranial magnetic stimulation (rTMS). Brain stimulation techniques generally have a number of different parameters which can be modified, and which could theoretically be tuned to improve treatment outcomes. These include physical parameters such as brain target and coil placement, as well as treatment protocol parameters such as length, intensity of treatment, and waveform used. Despite decades of research using stimulation techniques such as rTMS, we are only beginning to see clinical use of techniques which can tune some of these parameters in an individualized manner—for example, by using functional connectivity observed during fMRI to better define individualized brain targets (38)—but there is still a lack of understanding about the relationship between adjusting many of these parameters and treatment outcomes (41). We argue that the metaverse may be in a unique position to both help optimize clinical practices such that these parameters are set correctly and in a reproducible way, and also to provide practical and accessible data to help produce algorithms which may assist in the personalization of parameters and in the monitoring and prediction of treatment outcome. Any discussion of the use of data from the metaverse for this purpose, however, must be paired with a discussion of the neurotechnology ethics (42, 43), which we will elaborate on later in this paper.

### 3.1. Improved non-invasive brain stimulation targeting

**Figure 1:**
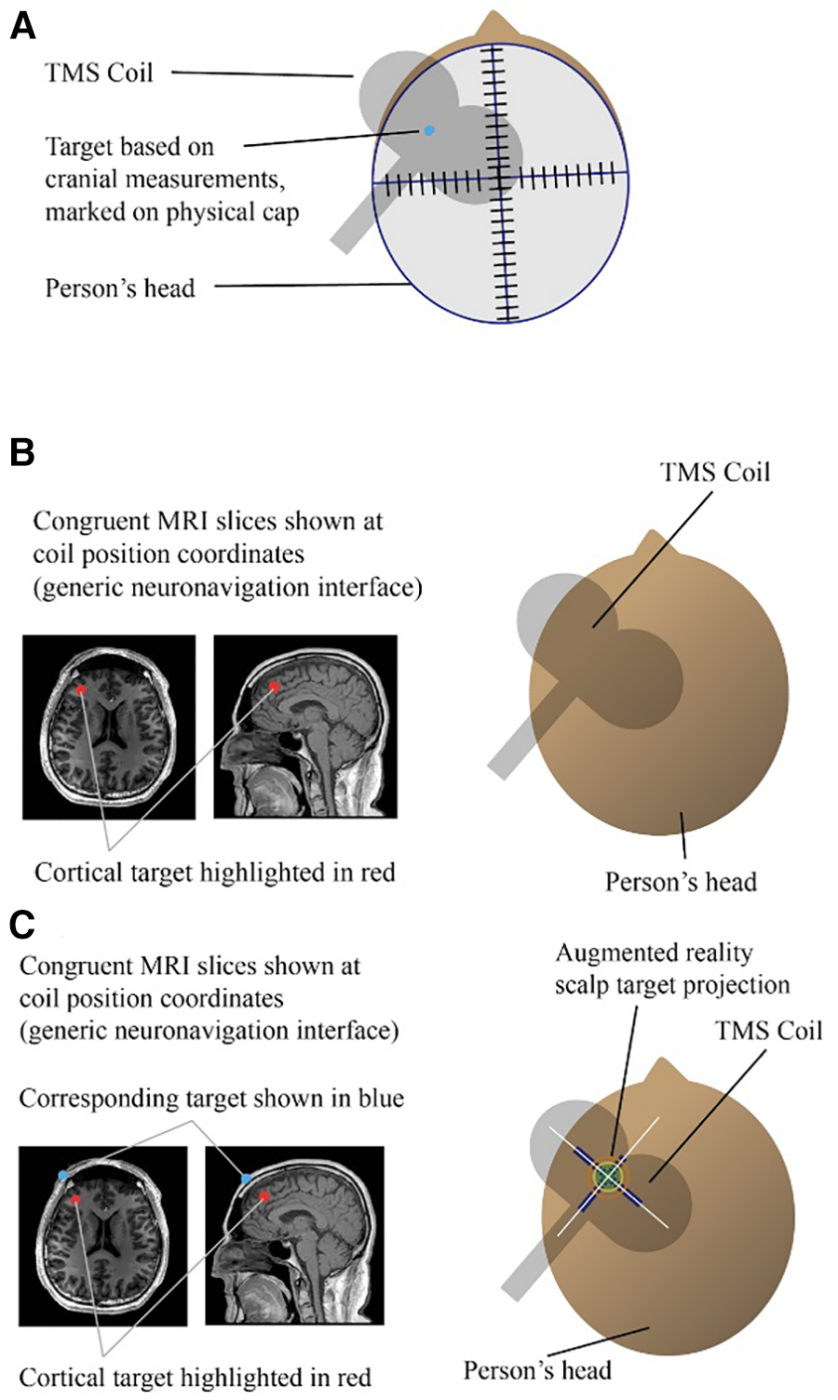
The top image (**A**) shows an example of doing rTMS targeting using only scalp based measurements and a cap based method. In this method, the center of the coil is placed over a target marked physically on a cap. The middle image (**B**) shows an example of doing rTMS targeting using a traditional neuronavigation method. In this method, the center of the coil is placed over a target digitally represented only on a computer monitor. The bottom image (**C**) shows an example of doing rTMS targeting using an AR neuronavigation method. In this method, the center of the coil is placed over a target that is digitally represented by a computer monitor and also virtually represented to appear as if it were on the patient's head (73, 74).

As previously mentioned, current forms of targeting utilized in brain stimulation include individualized functional-connectivity targeting through fMRI scans for rTMS (38) as well as deterministic tractography for surgical targeting in treatment-resistant depression (44). Augmented reality considers both digital and real-world applications made up of four types of markerless augmented realities: projection-based AR, location-based AR, overlay AR, contour-based AR, and separately a marker-based AR (ex. Figure 1. This is one example of how AR might look). Is it possible to leverage the metaverse and improve psychiatric care with augmented reality neuronavigation to improve targeting in neuromodulation treatments like TMS?.

Some modalities that have been proven highly effective rely on precision-targeting algorithms (38) which consequently require highly precise positioning practices. However, current methodologies for the precise, functionally-informed individualized physical placement of neuromodulation devices, such as rTMS coils, involve complex workflows and substantial training across multiple counterintuitive systems that add time to treatment sessions and require highly-trained technicians (45), potentially hindering the rollout of these advanced treatments in the clinical world. Essentially, such protocols require clinicians to place a sophisticated device about the size and weight of a brick (this is one way to understand the physicality of a TMS coil) within millimeter precision of an internal cortical target from viewing a person's hair and scalp alone, or, in well-resourced settings, congruent MRI slices tracked through neuronavigation software. In the future, the process could be refined and highly improved by, for instance, overlaying specific individualized targets *via* AR technologies like Google Glass in real, 3-D space, allowing clinicians to have visual markers in direct view of the patient's head, providing real-time feedback for closed-loop targeting, facilitating the delivery of highly precise stimulation.

Research has already shown evidence of the reliability and accuracy of AR-based targeting technologies. For example, the approach used in Sathyanarayana et. al, 2020 found that using marker-based mixed-reality models for targeting purposes on a model head was found to be within acceptable range (<5 mm variability) for accurate rTMS stimulation (45). Though the model was only accurate for maintaining accurate target markers after small rotations, this research offers promise that with further development and refinement, this application of AR for improved targeting precision may have the potential for future clinical use. Within the burgeoning field of noninvasive neuromodulation, leveraging technologies like overlaid augmented reality paradigms for precision treatments could be a highly effective method to decrease clinician-burden, treatment times, and unintentional stimulation site variability all the while facilitating widespread clinical implementation of advanced, highly-effective protocols.

### 3.2. Novel targeting algorithms and treatment response biomarkers in the metaverse

A recent trend in psychiatric research has been the search for measurable predictors and indicators of treatment response. These have classically included genetic, neuroimaging, and other biological markers alongside more classical questionnaire and cognitive assessment data (46). Despite significant efforts in this domain, regular use of biomarkers remains the exception in psychiatry, and more complex or expensive markers such as imaging and genetic data may not be widely accessible in the near future (46). As a result, and coincident with the growing availability of smartphones and other digital devices such as smart watches with integrated biological sensing functions, some researchers have turned their attention to so-called digital phenotyping (47), with the hope that the data produced by digital technology use, since it mediates so much of life in advanced economies, will contain the insights needed to better personalize treatment. While there have been some promising studies in this domain using various sensor types, there is still a significant gap between research and clinical implementation, and the evidence for the ability of specific features derived from digital sensors to accurately map to mental states remains limited (48). While this research has not yet provided definitive digital biomarkers which have made the leap to regular clinical practice, it has introduced these kinds of measures to psychiatry and, importantly, helped to develop the methodologies—such as artificial intelligence and related approaches—needed to analyze this kind of complex time-series data. Indeed, recent work has shown some promise in using machine learning to predict treatment outcome using measures that could be collected *via* digital apps, such as questionnaires (49–51). The promise of digital phenotyping is that it may generate large amounts of data without requiring the need for clinicians or patients to remember to complete assessments and collect data continuously in contexts which are relevant to real patient functioning, such as at work and during sleep. Despite these potential benefits, one potential limitation of current digital phenotyping approaches is that they rely on the measurement of behavior through an interface—the phone or watch—which does not necessarily record context about current activities; this context in turn may be necessary to train models that can reliably approximate a patient's mental state. In addition, one benefit of current digital phenotyping—its passive nature—also means that there are limitations on using it to generate standardized functional assessments where the goal is to determine what a patient can do, rather than what they are currently doing.

The metaverse may enable fundamentally different and complementary measures to status quo digital phenotyping. The metaverse is intended to be a place where people work, socialize, shop, and otherwise interact in a quasi-embodied manner (that is, through the use of a digital avatar). As discussed in a recent paper (40), this creates two key novel opportunities for measurement. The first is measurement of realistic behavior, especially social interactions in multiple contexts. This direct measurement of social behavior *in vivo* would be a novel feature of the metaverse. Voice, gaze, movements, and the length, quality, frequency, and overall flow of interactions in work and play environments could be captured for analysis, as well as the manner in which people complete all sorts of tasks. Having not just the correlates of the behavior, but the fullness of the behavior itself and its context for analysis may be a significant improvement in terms of the quality of the data from which digital biomarkers could be extracted. Indeed, one can imagine that digital biomarkers may be extracted on a patient-by-patient basis, hearkening back to the common clinical scenario where a clinician and patient may identify a patient's “warning signs” for relapse. The second novel measurement opportunity is through standardized functional assessment. Using the metaverse and appropriate editing software, a whole range of more ecologically valid functional assessments could be generated which could then be administered easily and affordably to patients depending on the clinical context—even patients clinicians may not have physical proximity to. This kind of assessment would be of significant benefit to clinicians who often may not have a clear picture of a patient's strengths and weaknesses in the functional domain.

But how does all this relate to brain stimulation? As discussed above, brain stimulation generally involves a number of parameters which could be modified, but learning which parameter to modify for a given patient relies on having a large body of data on relevant predictors. We posit that both passive and active measurement using the metaverse may provide this corpus of data, and then serve as a continual measure of treatment response which could be used as signals which could in turn train machine learning models aimed at helping to fine-tune treatment as it progresses. Indeed, it may even be possible to use measures and assessments in the metaverse to learn how to target specific symptoms using brain stimulation in order to provide relief in line with patient priorities. The nature of brain stimulation such as rTMS- its flexibility in terms of parameters to tune; its mechanisms of action tied to specific brain circuits; and its general safety and tolerability (52) makes it the perfect candidate when it comes to developing treatment prediction and treatment management algorithms using data from the metaverse. As such, algorithms could be developed to finely tune rTMS parameters during the course of treatment, adapting the treatment as the patient responds or fails to respond and performing this adaptation based on the continuous stream of functional data provided by the metaverse.

## 4. Biofeedback in the metaverse

Biofeedback is a self-regulation training technique that enables patients to volitionally control their psychophysiological state by understanding elements of their biological responses (53). In other words, biofeedback works by providing the user with insightful real-time feedback of their present physiological state. By attending to the feedback, one can manipulate the source of the signal, thereby altering their biological state. With practice, this process of biofeedback can enable someone to regulate their own psychophysiological states and responses. As such, biofeedback training can be an important component, or adjunct, to treatment in many disease states (54), and patients can learn to overcome adverse reactions to various stressors by simply focusing on otherwise subconscious physiological metrics such as heart rate, respiratory rate, or temperature (55). Additionally, the ability to volitionally manipulate VR environments provides a novel method to explore a multitude of psychophysiological states to inform and personalize treatment parameters for patients.

In psychiatric disease states, a patient's ability to use biofeedback depends on recapitulating specific scenarios to trigger unwanted, intrusive, or painful thoughts or feelings. This is not always achievable and can limit the effectiveness of any biofeedback therapy. Virtual and augmented reality technology provide a unique capability to model and manipulate immersive real-world environments. This capability may lend itself very well to providing tailored and individualized, patient-centric approaches to recreate stressors while at the same time providing the ability to monitor and visualize such physiologic responses as pupil size or heart rate (37). Imagine, for instance, a real-world scenario of a psychiatric patient feeling extremely anxious or panicked; an AR based biofeedback device could provide the patient with visual indicators of their heart rate, respiratory rate, temperature, or even pupil dilation. This feedback can then be used as a signal for self-regulation by the patient in real-time to help them manage their anxiety. This scenario could also be envisioned prophylactically in an immersive VR based environment.

Conventionally, biofeedback would take place in a doctor's office equipped with the necessary sensors and hardware. However, with wearable technology becoming more ubiquitous (ex. Smart phones, smart watches, and other smart sensors) biofeedback has become increasingly available for at-home and remote use. Now, with the addition of XR/metaverse technology, biofeedback is not only available remotely, but it is available with extreme fidelity. Although XR technology is not accessible to all, its ability to be portable makes it possible to offer psychiatric patients high fidelity biofeedback therapy in areas where access to mental health professionals is limited. The following section of the paper will discuss some of these limitations surrounding equitable access to the metaverse in the context of psychiatric care.

## 5. Discussion

### 5.1. Concerns regarding accessibility and equity of the metaverse in psychiatry

As new technologies emerge, it behooves us to consider the ethical implications, particularly in the realm of accessibility and equity (56). The metaverse and its requisite technologies (AR/VR/MR) have the potential to reshape much of our world (57). Yet fundamental elements, like access and accessibility, can often be overlooked in the initial creation of a product or innovation, and lost in the wake of popular reception, potential impact on the status quo, and rapid efforts to commercialize. Unless explicitly a venture in social impact, digital services are rarely designed to address the digital divide. Socio-economic barriers to entry, propagation of prejudices into the digital space, and access for individuals with disabilities are too easily relegated to afterthoughts (58). However, it's imperative that we consider the socioeconomic landscape technologies will enter before they have the chance to further contribute to marginalization, inaccessibility, and inequality in healthcare and beyond (59, 60).

Most clinical interventions' efficacies don't rely on a patient's technological literacy, nor do they necessarily require patients to be in possession of otherwise healthcare-unrelated gadgetry, like costly VR headsets. The utility of current treatments and other clinical interventions that require technologies like a smartphone, computer, or internet access hinge upon patients owning these devices or having access outside of their doctor's office (60). Will this be the same truth for clinical applications of the metaverse? If so, let us consider what this entails. Participating in the metaverse requires access to technologies such as a VR headset or a smartphone and VR-compatible headgear, and, depending on the application being run, access to a high-end computer. Access to high-speed internet is also required to enter the metaverse. We already live in a world where access to indispensable pieces of technology like the internet is inequitable. According to the United Nations Telecommunications report as of 2021, 2.9 billion people worldwide do not have stable internet access, with 96% representing those living in developing countries (61). As such, a significant part of the global population would not be physically able to access the metaverse at present.

Even within areas and communities where high-speed internet access is commonplace, a substantial portion of even that subset of the population will likely not be able to afford the specialized devices required for metaverse access. VR headsets costs range from several hundreds to over a thousand dollars. In the United States, a country where roughly 97% of the population has access to high-speed internet (62), 24% of consumers had no emergency savings at all with another 39% of consumers having less than a month of income saved in 2022 (63). There already exist layers upon layers of increasingly difficult-to-reach rungs of a ladder, at the top of which lie our most groundbreaking innovations. How can we expect the population to access virtual reality equipment when access to basic elements, like internet access (especially at speeds required for modern applications like the metaverse), are not guaranteed? Will the metaverse and its clinical applications be designed only for those who have had a hand in their creation? Or will they be inclusive innovations that aim to ameliorate and address the non-meta world's solidly embedded biases, injustices, and inequalities? Will the patient-facing clinical applications of the metaverse become another piece of technology available not to the average person seeking psychiatric care, but solely another intriguing high-end product for the biohacking elite?.

These foreseen shortfalls in access can be divided into two groups: the gap between those that can and cannot access these means of care, and then the gap that exists within the population that is able to access these technologies. This can be particularly jarring when we consider the potential clinical benefits that metaverse technologies have to offer. Within the subset of users who are able to access the metaverse, those who are not familiar with XR technology may struggle to navigate and participate, affecting the practical use and efficacy of such interventions. If individual digital literacy has a hand in determining efficacy of said interventions, disparities in digital literacy at both the individual and group level might greatly impact treatment outcomes, exacerbating existing health inequalities between better-educated, affluent populations and less well-educated and lower socioeconomic status (SES) groups (56, 57).

In the physical world we are not naive to the many prejudices that exist (based on race, sex, class, able-bodiedness, etc. to name a core few). While we may strive to acknowledge, amend, and reduce future harm and inequality, the metaverse reflects *current reality*, and will therefore reflect these same biases and inequalities unless direct action is taken to address and remedy (64). Discrimination and prejudice in the metaverse may replicate or even amplify existing biases and inequalities (56, 57, 64). It may allow people to interact anonymously and without consequences, acting as a new medium for prejudice and discrimination (64). Because of this, representation of different cultures, identities, genders, and abilities is incredibly important. It supports the metaverse reflecting and representing the diversity of its users and the greater global community at large. One example is the digital representation of self—the avatar. Evidence suggests that avatars that resemble their users lead to higher reported self-presence and realism than users embodied as uniform avatars (65). In clinical applications of the metaverse, realism and self-presence may prove to be significant in treatment outcomes, specifically in disciplines like psychiatry (66). Allowing users to authentically represent themselves in the Metaverse with phenotypic variety that spans across identities could be a simple and effective solution to support and encourage diversity, inclusion, and belonging. Similarly, the metaverse should aim to recreate environments that are culturally and geographically sensitive to its users to be considerate and accommodating of their unique backgrounds. In principle, a psychiatric patient should be able to pick an environment that is representative and comfortable for them, which may provide a more equitable experience and better treatment outcomes in the context of psychiatric care.

Entities investing in and shaping these technologies must take robust action to create spaces of belonging for users of all identities and backgrounds, including those of varying abilities. Though the possibility of using XR for rehabilitating cognitive and motor disabilities is indeed on the horizon (67), we must acknowledge that virtual experiences en masse are not necessarily designed with accessibility in mind. In the instances outside of use-cases for sensory, cognitive, and motor rehabilitation of which there will be many, users who have physical disabilities or lost or diminished sensory abilities such as impaired vision, audition, or proprioception may require specialized hardware to participate equally in the metaverse. Without significant action aimed at inclusive design, this can be an additional barrier for access for users on top of the financial and socioeconomic hurdles that already obscure and limit access to healthcare and technology (68). It is imperative that the creators and regulators of these immersive technologies consider these issues to ensure that the virtual world is accessible and equitable for all users (56, 57, 64). We bring forth these concerns to raise attention to potential pitfalls and challenges in creating accessibility, equitability, and unbiased clinical applications of these advanced technologies for the sake of just care and equal health outcomes. Oftentimes in society, it is those with least power to advocate who are denied access to new technologies and services, especially in healthcare: which is after all a human right (69).

### 5.2. Concerns regarding data security, privacy, and misuse of the metaverse in psychiatry

When discussing the use of data collected in the metaverse and its potential application to modulating the function of a brain stimulation device, or in biofeedback, whose purpose is to change or monitor a patient's mental state, we must consider issues of security, privacy, and data use (40). The metaverse will be supplied by private corporations who have, necessarily, a profit motive (42, 43). It is now common knowledge that certain companies which are engaged in building the metaverse had knowledge that instances of its predecessor technology, social media, was harmful in certain circumstances to the mental health of youth, and chose to keep this information secret (see Benrimoh et. al, 2022, for a discussion). Some of these same companies have been involved in scandals which saw user data being harnessed to modify beliefs and sway elections (70).

As such, we argue that it should be assumed that the data collected by the metaverse which can be used for medical purposes could also be used for less noble causes, such as shaping consumer beliefs and behavior. While this would most likely occur *via* currently employed techniques, such as targeted advertising, the chance that manipulation of patients through commercially available brain stimulation or biofeedback devices may one day be possible should not simply be discounted (43). We also argue that we should assume that abuses will occur unless rather explicit limitations are placed on what can legally be done with data collected in the metaverse which is used for a medical purpose. In line with our perspective on accessibility and equity, new technologies often first develop rapidly without oversight and regulation and only retroactively are the necessary regulations put in place. We contest that it is imperative to intentionally design and implement robust regulations in tandem with the design and implementation of the metaverse in psychiatric care. In a recent paper discussing this, Benrimoh et. al, (2022) noted that metaverse applications created with a clear medical purpose should in theory already fall under the jurisdiction of medical device regulations, and that data collected in these contexts should therefore be handled in line with relevant data protection legislation, such as HIPAA in the United States. or PIPEDA in Canada. Metaverse applications not created with a purely medical context should be required to generate standardized reports to be used by researchers in order to determine their impact on mental health during post-marketing and beta testing, with the aim of determining which elements of the metaverse require further regulatory attention.

Beyond misuse of big data collected from clinical applications of the metaverse being used for third-parties to profit, the information that is collected and stored by healthcare providers is highly sensitive and personal in nature. Like in any healthcare setting, unauthorized disclosure of this information can have serious consequences for the individuals concerned. If someone's medical information is made public without their consent, it could lead to embarrassment, discrimination, or even financial harm. Therefore, protecting patients' privacy is essential for building trust between patients and healthcare providers, and a lack of trust or confidence in their confidentiality could have negative impacts on patients' mental health and well-being. With the increasing use of electronic health records, protecting digital privacy in the healthcare industry becomes even more relevant (71). Especially given the highly sensitive nature of potential psychiatric applications of AR/VR, without assurance and confidence that their personal information (ex. experiences, behavior, and biometric data in the metaverse and clinical applications) is secure, it would be reasonable for patient populations to be less likely to seek treatment or share important information with their healthcare provider when using these technologies.

Because of the significance of privacy and security of patient data, it's imperative to consider how it can become compromised; cyber attacks and data breaches are two core examples. Data breaches are security incidents in which sensitive, protected, or confidential data is accessed, disclosed, or otherwise compromised by an unauthorized individual or entity. Such breaches can occur through various means, such as hacking, phishing, malware attacks, or insider threats. XR systems typically involve the use of unique, sophisticated hardware and software, and as such, they may be especially vulnerable to hacking or other forms of cyber attack. Without robust security measures in place to secure patient data across platforms, this could lead to the theft or unauthorized access of sensitive patient information, which could have serious consequences for both patients and healthcare providers (72).

## 6. Conclusion

The metaverse, or Web 3.0, poses many exciting new possibilities for Psychiatry 3.0. Future medical education programs should consider the ways in which XR technology can be leveraged in the metaverse to optimize the training of new physicians, especially those in medical specialties where physical examination is not paramount, like psychiatry. Psychiatrists should also be aware of the development of the metaverse/XR and how it will integrate into psychiatry to reshape the way psychiatric care is offered; where brain stimulation and biofeedback are but a few key areas where XR may improve efficacy. We expect to see significant growth in the XR and metaverse commercial industry in the coming years which will surely surge the medical and psychiatric interest of leveraging the metaverse to optimize patient care. Although exciting, moving any psychiatric care into the metaverse raises a number of ethical and safety challenges that ought to be addressed. Therefore, as society takes this step forward, we urge all responsible parties (educators, doctors, patients, lawmakers, software/hardware developers, and corporate entities) to remain cautious and conscientious to ensure that the metaverse is a safe, accessible, just, and equitable environment for all of its users. We have the opportunity to build this digital society from the ground up; let us build it in a way that puts the needs of people first.

## Data Availability

The original contributions presented in the study are included in the article/Supplementary Material, further inquiries can be directed to the corresponding author.
